# Blood–Brain Barrier (BBB) Dysfunction in CNS Diseases: Paying Attention to Pericytes

**DOI:** 10.1111/cns.70422

**Published:** 2025-05-15

**Authors:** Tianrui Yu, Zixuan Wang, Yanghang Chen, Yuanyuan Xiang, Moxin Wu, Manqing Zhang, Xiaoping Yin, Zhiying Chen

**Affiliations:** ^1^ Department of Neurology, School of Clinical Medicine Jiujiang University Jiujiang China; ^2^ Jiujiang Clinical Precision Medicine Research Center Jiujiang China; ^3^ Department of Medical Laboratory Affiliated Hospital of Jiujiang University Jiujiang China; ^4^ Jiangxi Provincial Key Laboratory of Cell Precision Therapy, School of Basic Medical Sciences Jiujiang University Jiujiang China

**Keywords:** Brain–Blood Barrier, CNS diseases, Extracellular vesicle, Neuroinflammation, Pericyte

## Abstract

**Background:**

Dysfunction of the blood–brain barrier (BBB) is an important pathological mechanism in central nervous system (CNS) diseases and can trigger a series of pathological reactions, such as neuroinflammatory responses, oxidative stress, immune infiltration, etc., thereby worsening brain damage. However, pericytes are often overlooked by researchers, and no review research has yet summarized the mechanism by which pericytes contribute to BBB dysfunction in CNS diseases.

**Results:**

Therefore, this review explores the pathophysiology of BBB dysfunction in CNS diseases and provides a detailed account of the biological characteristics of pericytes, especially the controversy over their biomarkers. Subsequently, we review the role of pericytes in CNS diseases such as Alzheimer's disease, vascular dementia, multiple sclerosis, ischemic stroke, and hemorrhagic stroke, with a particular focus on the role of pericytes in BBB dysfunction. In addition, we also discuss treatments based on pericytes, such as regenerative medicine that induces pericyte differentiation and Pericyte‐Extracellular Vesicles.

**Conclusions:**

This review aims to provide a more comprehensive understanding and guidance on the role of pericytes in BBB dysfunction in CNS diseases and serve clinical treatment.

## Graphical abstract

This review highlights the critical role of pericytes in BBB dysfunction in central nervous system diseases. It explores their involvement in diseases like Alzheimer's, stroke, and multiple sclerosis, and discusses potential therapeutic strategies, including regenerative medicine and PC‐EVs.

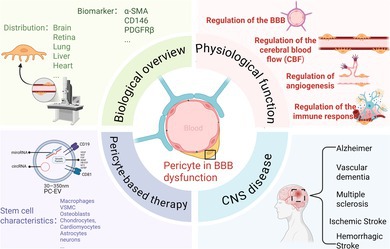

**Abbreviations**
ABCC9: ATP‐binding cassette transporter subfamily C member 9
ad
: Alzheimer's disease
Aβ: amyloid beta
BBB: blood–brain barrier
BEC: brain endothelial cells
BM: basal membrane
CBF: cerebral blood flow
CCH: chronic cerebral hypoperfusion
CD146: melanoma cell adhesion molecule
CNS: Central nervous system
CSPG: chondroitin sulfate proteoglycan
EAE: experimental autoimmune encephalomyelitis
EC: endothelial cell
EV: extracellular vesicle
GPCRs: G protein‐coupled receptors
HIF‐1: hypoxia‐inducible factor‐1
ICH: intracerebral hemorrhage
IgG: immunoglobulin G
IL: interleukin
IS: ischemic stroke
LRP‐1: LDL receptor‐related protein‐1
LRP1/apoE: low‐density lipoprotein receptor‐related protein 1/apolipoprotein E
MCP‐1: monocyte chemoattractant protein‐1
MHC II: major histocompatibility complex class II
MMP: matrix metalloproteinase
MS: multiple sclerosis
NVU: neurovascular unit
PC‐EV: pericyte‐extracellular vesicle
PDGFRβ: platelet‐derived growth factor receptor beta
ROS: reactive oxygen species
SCI: spinal cord injury
TGF‐β: transforming growth factor‐beta
TJPs: tight junction proteins
TMEM16A: transmembrane protein 16A
TNF‐α: tumor necrosis factor‐alpha
VaD: vascular dementia
VEGF: vascular endothelial growth factor
vSMCs: vascular smooth muscle cells
ZO‐1: zonula occludens‐1

## 1 Introduction

The blood–brain barrier (BBB) is a cell interface between the blood and the central nervous system (CNS) that plays a key role in maintaining CNS homeostasis. BBB dysfunction is an important pathological factor in CNS diseases. Some studies have shown that BBB dysfunction in CNS diseases can trigger a series of pathological reactions, such as immune cell infiltration, oxidative stress, and insufficient cerebral blood flow, which can further aggravate brain damage [1].

Pericyte, as one of the components of the BBB, is a multifunctional cell with paracrine properties, stem cell properties, and contractility, and is thought to play an important role in a variety of biological processes [2]. However, pericytes are often overlooked in the study of neurovascular units (NVU). Recent studies have shown that the loss or dysfunction of pericytes is closely related to a variety of CNS diseases, including Alzheimer's disease (AD), vascular dementia (VaD), ischemic stroke (IS), intracerebral hemorrhage (ICH), and multiple sclerosis (MS) [3]. Moreover, studies have shown that Pericyte‐Extracellular Vesicles (PC‐EV) may slow the progression of CNS diseases [4].

Overall, the biological characteristics of pericytes indicate that they have the potential to become a new target for the treatment of CNS diseases. A deeper understanding of the complex interactions between pericytes and other components of the NVU is important for the development of new treatment strategies that target the recovery of the BBB and the function of pericytes to reduce CNS damage and improve treatment outcomes.

## 2 BBB Dysfunction in CNS Diseases

BBB is a structure composed of pericytes, brain endothelial cells (BEC), astrocytes, cell junctions, and a basal membrane (BM) that protects the CNS from toxic and harmful substances in the blood and allows water‐soluble nutrients and metabolites to enter the CNS [1, 5]. In addition, leukocyte adhesion molecules are essential for leukocytes to cross the endothelium and enter the CNS. However, due to the low expression of leukocyte adhesion molecules in the BEC, this limits the interaction between immune cells and endothelial cells, effectively preventing the infiltration of immune cells into the healthy CNS [6, 7]. In addition, BBB is also a key component of the NVU, which is a structural unit composed of vascular endothelial cells, pericytes, astrocytes, neurons, and other related cells. Together, they regulate cerebral blood flow (CBF), remove metabolic waste, and participate in immune responses [8]. BBB dysfunction is an important pathological feature of CNS diseases, characterized by the loss of its structural integrity and normal function. When the BBB is damaged, harmful substances that were previously blocked, such as bacteria, viruses, immune cells, and macromolecules, are able to enter the brain, breaking the immune isolation of the brain and triggering a series of pathological reactions, thereby exacerbating brain damage [9]. CNS diseases with BBB dysfunction as the main pathological feature include AD, VaD, IS, ICH, MS, etc. [10, 11, 12]. In neurodegenerative diseases, BBB dysfunction often occurs before clinical symptoms appear. In AD, the accumulation of Aβ protein can lead to an inflammatory response and cytotoxicity, which in turn leads to BBB dysfunction [13]. In the early stages of VaD, long‐term chronic cerebral hypoperfusion (CCH) can lead to a decrease in tight junction proteins ZO‐1, occludin, and claudin‐5, resulting in BBB dysfunction [14]. After the ischemic stroke occurs, it can lead to BBB dysfunction, causing pericyte immune cells to infiltrate into the brain and mediate an inflammatory response, which in turn exacerbates the pathological events of ischemic stroke and leads to more severe brain damage [10]. In multiple sclerosis, dysfunction of the BBB can lead to infiltration of immune cells into the CNS, causing demyelinating lesions that severely affect nerve conduction velocity [15]. In summary, BBB dysfunction is an important pathological basis for the development of various CNS diseases. The deeper research into the mechanisms of BBB dysfunction is of great significance for understanding the pathogenesis of CNS diseases and developing new treatment strategies.

## 3 Overview of the Biology of the Pericyte

### 3.1 Distribution and Origin of the Pericyte

In 1874, “Rouget cells” were first described as contractile cells surrounding the endothelial cells of small blood vessels [16]. After that, Zimmermann called this type of cell close to the endothelial cell as pericyte [17]. After 150 years of exploration, pericytes are considered to be a kind of multifunctional cell that surrounds the endothelial cells in the lumen of capillaries and arterioles and shares a basement membrane with the endothelium. The density of pericytes in the cerebral cortex of the CNS (one pericyte for every 3–5 endothelial cells) is much higher than in skeletal muscle (one pericyte for every 10–100 endothelial cells) [18]. Brain pericyte cells are therefore essential for maintaining the homeostasis of the CNS and a lack of brain pericyte can lead to serious neurological disorders. In the CNS, the origin of pericyte cells seems to be related to their anatomical location. Previous studies have shown that in the zebrafish model, pericyte cells appear at the 52nd hour after fertilization and develop together with the brain and trunk vasculature on the second day of development [19]. Quail‐chick chimera experiments have demonstrated that forebrain pericytes originate from neural crest cells, whereas pericytes in the midbrain, brainstem, and spinal cord are derived from mesodermal mesenchymal stem cells [20]. Moreover, in vitro experiments have shown that pericyte‐like cells can be generated from hESC (Human embryonic stem cells) and iPSC (Induced pluripotent stem cells) via the mesoderm (mPC) or neural crest (ncPC) pathways [21]. Furthermore, studies have shown that pericytes derived from the mesoderm can differentiate into CD274‐positive capillary pericytes with proinflammatory phenotypes and DLK1‐expressing arteriolar pericytes exhibiting contractile properties [22]. The previous experiments provide strong evidence for the idea that the origin of pericyte cells seems to be related to their anatomical location. Although the embryonic origin of pericyte cells has been fully understood, little is known about how pericyte cells (especially pericyte cells in the brain) function in the brain under physiological conditions. The function and mechanism of these cells need to be explored in more depth in the future.

### 3.2 Anatomical Location and Morphological Characteristics of the Pericyte

**FIGURE 1:**
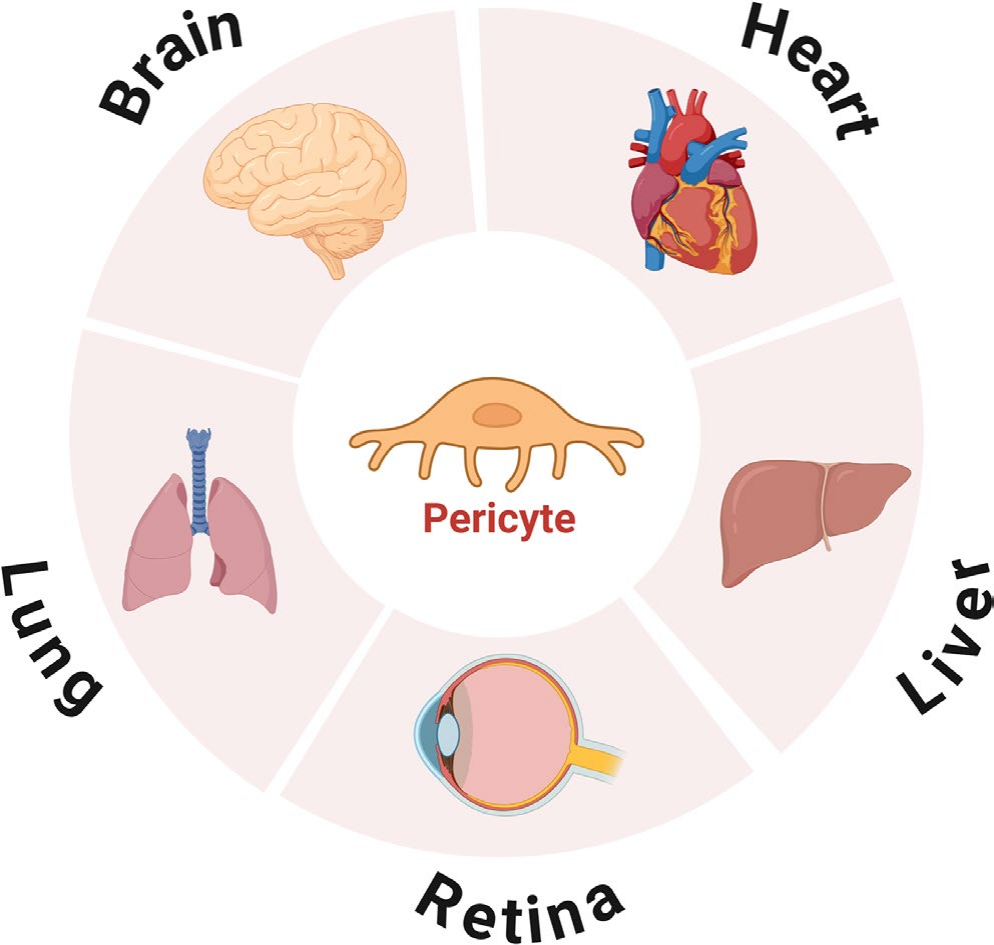
Organs in which pericytes are distributed.

Thanks to the advent of the electron microscope, the PC was defined as a cell embedded in the basement membrane (BM) of blood vessels and wrapping around capillaries, in close contact with endothelial cells [23]. However, pericytes exhibit different morphological characteristics in different tissues. In the kidney, pericytes are usually connected to microvessels (e.g., renal tubular capillaries), and they appear as star‐shaped or spindle‐shaped cells surrounding the endothelial cells of capillaries [24]. Pericytes in the heart that contain α‐SMA are spindle‐shaped and mainly found at the arteriole‐capillary junction of the heart, which is considered to have the ability to control the coronary blood flow [25]. The pericytes of the liver are mainly hepatic stellate cells (HSC), which are located between the parenchymal cell plates and the sinusoidal endothelial cells. These cells play a key role in driving liver fibrosis and are associated with inflammation and cancer [26]. The pericytes of the retina are spindle‐shaped, with multiple cell protrusions. They are an important component of the blood‐retina barrier during retinal angiogenesis [27, 28]. In the lungs, the pericyte cells are mainly responsible for gas exchange between the lungs and the outside world [29]. Besides, lung pericytes can respond to lung injury by expressing various chemokines and can detect proinflammatory molecules released following epithelial barrier damage, participating in the recruitment of circulating leukocytes [30]. The brain pericyte cells are located at the NVU. The cell body is ovoid, contains a distinct nucleus, and has abundant cytoplasm and long thin protrusions [25]. Structurally, these protrusions are connected to endothelial cells and the basement membrane to form a complex network structure [2]. Functionally, these protrusions are the basis for the physiology of pericyte cells regulating blood flow and modulating the BBB [2] (See Figure 1 for details).

### 3.3 Identification of the Pericyte

The identification of pericytes is mainly based on their specific anatomical location and morphology or the combined use of multiple markers. Due to the special location of pericytes around the vascular endothelium and the currently generally accepted view that pericytes belong to the same lineage and cell type as vascular smooth muscle cells (vSMCs), pericytes are often confused with vSMCs, macrophages, and fibroblasts. Currently, there are no molecular markers that can be used to clearly identify pericytes. In addition, pericytes show significant heterogeneity in different tissues, organs, and physiological states. In other words, pericytes markers are dynamically expressed (upregulated or downregulated) in pericytes during different physiological states or developmental stages of the body [3]. This section summarizes the subtypes of pericytes and various biomarkers and aims to provide an important basis for the identification of pericytes (especially brain pericyte). The pericytes shows heterogeneity in different organs. In the CNS, the brain pericyte can be divided into three subtypes based on their morphology and distribution area: sheath‐like pericyte, reticular pericyte, and fine‐chain pericyte [31]. The results of high‐throughput single‐cell RNA sequencing (scRNA‐seq) showed that retinal pericytes are mainly composed of three functionally distinct Subtypes, including an activated pericyte Subtype with angiogenic capacity, a pericyte Subtype with an inflammatory response, and a resting pericyte Subtype with a homeostatic function [32]. Another classification of pericytes in skeletal muscle can identify two subtypes based on different expressions of Nestin, namely type 1 (Nestin‐GFP(−)/NG2‐DsRed(+)) and type 2 (Nestin‐GFP(+)/NG2‐DsRed(+)) pericytes [33]. Moreover, there are two subtypes of Higd1b‐positive pericytes in the lung: type 1 is located in the capillary network (diameter < 10 μm), and type 2 is located in the region between capillaries and arterioles (diameter < 25 μm) [34].

The most common markers for pericytes are mainly α‐smooth muscle actin (α‐SMA), neuron glial antigen (NG2), and platelet‐derived growth factor receptor‐β (PDGFRβ) [35]. The expression of α‐SMA protein varies in different pericytes subtypes. The expression of α‐SMA in cells of the true capillaries (midcapillaries) is lower than that in the transitional pericytes of pre‐ and postcapillary microvascular segments [36]. NG2 is expressed by pericytes in newborn microvessels, and NG2 expression is upregulated during immature cell movement or mitotic activity, while it is downregulated during cell maturation or quiescence [37]. Platelet‐derived growth factor BB (PDGF‐BB) is a factor involved in mitosis, migration, and survival. PDGF‐BB also recruits stable pericytes into blood vessels by trapping them in the extracellular matrix [38]. Recently, Higd1b has been shown to be a genetic marker for pericytes. Klouda et al. used single‐cell RNA sequencing to analyze Higd1b‐positive pericytes and found that there are two subtypes. Type 1 pericytes are in a resting state under hypoxic conditions, while type 2 pericytes upregulate the expression of vimentin and smooth muscle cell markers under hypoxic conditions, suggesting an important role in vascular remodeling [34]. To provide a visual understanding of pericytes markers, Table 1 lists the currently known brain pericyte markers.

**TABLE 1:** Summary of markers of brain pericytes.

Brain pericyte marker	Expression condition	Function	References
RGS‐5	Pericytes that acutely detach from the vascular wall after stroke express RGS5	Involved in angiogenesis and maintaining vascular stability	[39, 40, 41]
Endosialin (CD248)	Expression in brain pericytes cells and downregulated during development.	Promote angiogenesis in the fetus's brain	[42]
Fluoro‐Nissl dye NeuroTrace 500/525	NeuroTrace selectively labels non‐contractile pericytes located on capillaries	Suggests the existence of a molecular transport mechanism unique to pericytes	[43]
CD11b	Histones of neutrophil extracellular traps induce CD11b Expression in brain pericytes via dectin‐1 after Traumatic Brain Injury	Promotes neuroinflammation after brain injury	[44]
Neuron‐glial antigen 2 (NG2)	Expressing after ischemic stroke	Involved in regulation of vascular function	[45]
Tbx18	Expressing after ischemic stroke	Involved in regulation of vascular function	[45]
Potassium inwardly rectifying channel subfamily J member 8 (Kir6.1)	Expressing after being activated by adenosine signal	Involved in regulation of vascular function	[46]
Platelet‐derived growth factor receptor beta (PDGFRβ)	Generally expressing in pericytes	Pericyte proliferation and recruitment involved in the process of angiogenesis	[47]
Aminopeptidase N (CD13)	Expressing in the cranial neural crest‐derived pericyte‐like cells (hPSC‐CNC PCs)	Participate in the regulation of vascular function	[48, 49]
Desmin	Expressing in that repair of spinal cord injury	A structural protein	[50]
Alpha‐smooth muscle Actin (α‐SMA)	Not expressed in pericyte from mouse embryonic brain and human brain; expressed in pericyte from chicken embryonic brain	A structural protein; important for contraction of pericyte and regulation of blood pressure	[31]
Melanoma Cell Adhesion Molecule (CD146)	Expressing with the increase of pericytes coverage	Promote the formation of BBB	[51]
ATPase 13A5 (Atp13a5)	A specific marker	May be related to the establishment of BBB	[52]

In summary, the heterogeneity of pericytes and the lack of clear molecular markers make it challenging to study them in different tissues, organs, and physiological states. In‐depth research on pericytes markers provides potential targets and strategies for revealing their role in different physiological and pathological conditions and for the clinical diagnosis and treatment of CNS diseases.

## 4 The Physiological Function of the Pericytes in the CNS

### 4.1 Regulation of the BBB

Although the endothelial cells of the brain capillaries are the main cell component of the BBB, pericytes play a significant role in the formation, stability, and functional regulation of the BBB [53]. Recently, studies have shown that lactate produced by endothelial cells through glycolysis is transported to pericytes via MCT1/5. At the same time, it was found in endothelial‐specific Glut1 KO mice that endothelial cell GLUT1 loss leads to pericyte apoptosis and BBB breakdown. Therefore, lactate of endothelial origin may be key to maintaining pericyte function and regulating BBB permeability [54]. However, the regulation of BBB permeability by pericytes appears to be heterogeneous. In *pdgf‐b* ^*ret/ret*^ mice, pericytes are lost and BBB permeability to endogenous macromolecules is increased in the cortex, hippocampus, and striatum, while it is significantly decreased in the diencephalon, midbrain, and cerebellum [55]. There are currently two possible explanations for this phenomenon. One is that the pericytes in different brain regions may lead to functional differences, which in turn affect the permeability of the BBB [20]. Second, in addition to pericytes, there may be other unknown regulatory pathways involved in BBB permeability. During embryonic development, pericytes are also essential for the formation of the BBB, and the stage of BBB formation corresponds to the stage of pericyte recruitment, which precedes the stage of astrocyte generation [53]. Not only that, BBB function is established in mice starting on day 15.5 (E15.5), and MFSD2A expression is reduced in mice with a pericyte defect, leading to increased transcellular transport of endothelial cells and damage to the integrity of the BBB. Interestingly, however, MFSD2A−/− mice have impaired BBB function but normal vascular network development, suggesting that vascular development and BBB formation are independent of each other [56]. Therefore, MFSD2A may be a potential therapeutic target for BBB recovery, and future treatments can focus on restoring BBB permeability without having to rely solely on reconstructing the vascular network. Furthermore, Chen et al. found reduced expression of the tight junction protein claudin‐5, which is responsible for maintaining BBB integrity, and BBB breakdown in CD146‐specific KO mice [57]. In contrast, CD146 knockout in pericytes significantly reduces pericyte coverage and damages BBB integrity. At the same time, pericytes promote BBB maturation by inhibiting endothelial CD146 mRNA and protein expression through TGF‐β negative feedback [51]. Not only that, CD146 is also involved in the process of PDGF‐B/PDGFRβ‐induced pericyte recruitment. It regulates this signaling pathway in various ways, including self‐dimerization, binding to PDGFRβ, recruiting PI3K, and binding to the cytoskeleton [58]. In summary, CD146 is important for the BBB, but previous studies have only focused on its role in BBB development. The role of CD146 in the repair process after BBB damage (such as ischemia–reperfusion and neuroinflammation) still needs to be further explored. In addition to during development, pericytes in adulthood and aging are also important for maintaining the BBB.

### 4.2 Regulation of the Cerebral Blood Flow (CBF)

A lack of CBF is the pathophysiological mechanism of many CNS diseases (e.g., Alzheimer's disease, ischemic stroke, and vascular cognitive impairment). For example, a lack of CBF is a central factor in the occurrence and development of ischemic stroke, and a lack of CBF after ischemic stroke can lead to pathophysiological processes such as calcium ion influx, BBB damage, neuroinflammation, and cell death, thereby aggravating brain damage [59, 60, 61]. In the early stages of AD, CBF in certain areas of the brain decreases by about 50%. Moreover, studies have shown that the reduction in CBF mainly accelerates the progression of AD by upregulating the BACE1 enzyme, which produces Aβ, and promoting the overphosphorylation of tau [62, 63, 64]. Therefore, it is important to determine the role of pericytes in regulating CBF. Due to the embryological similarity of pericytes and smooth muscle cells (SMC) and their anatomical location within the basement membrane of capillaries, many people believe that pericytes regulate blood flow at the capillary level. In fact, in vitro studies using rodent brain slices have first demonstrated that pericytes can change capillary diameter in response to electrical stimulation, neurotransmitters, and ischemia [65]. In vivo experiments, however, have produced contradictory results [66, 67]. However, using optogenetic techniques, Hall et al. showed that the relaxation of smooth muscles can lead to capillary dilation and that contractile cells surrounding brain capillaries control the blood supply to healthy neurons [68]. Nevertheless, a study rejected the idea that “pericytes participate in CBF regulation.” Hill et al. found that optogenetic stimulation, physiological nerve activation, and diffuse depolarization of pericytes for about ten seconds could not cause vasoconstriction or vascular dilation. Moreover, optogenetically induced vasodilation only occurred in vessels covered with smooth muscle [66]. The photosensitive channel rhodopsin‐2 (ChR2) is a non‐selective cation channel permeable to sodium, potassium and calcium and it can be opened and depolarized by 488 nm light stimulation [69, 70]. Some studies have shown that ChR2 activation can lead to slow contraction of the pericytes, followed by contraction of the underlying capillaries, resulting in a decrease in capillary diameter of about 8% and a decrease in RBC velocity, thereby regulating capillary blood flow in the brains of aging mice [71]. Interestingly, Hartmann et al. found that the effect of pericytes on CBF is very slow, unlike the classic rapid regulation of small artery smooth muscle. In addition, after the optical ablation of pericytes, the capillaries undergo selective local dilation and redoubled blood cell flux [72]. Cyclic cell shrink is controlled by the interaction between Ca^2+^ and L‐type voltage‐gated calcium channels (CaVs) and the Ca^2+^‐gated Cl^−^ channel TMEM16A (also known as ANO1, which is a member of the membrane‐associated protein family). This view is also confirmed in AD model mice, and oral administration of nimodipine can improve the early reduction in CBF in AD mice [73, 74]. Therefore, TMEM16A may be a therapeutic target for improving the reduction in CBF in CNS diseases [74, 75, 76]. In the future, a specific regulator of TMEM16A channel activity can be developed to address the problem of reduced CBF. Not only that, but it is also controversial whether pericytes in ischemic environments affect CBF. Some studies have shown that pericytes are sensitive to ischemic environments and contract for a long time after ischemia, leading to local retention of red blood cells [77]. However, studies have also shown that under ischemic conditions, the smooth muscle of the arterioles constricts rather than the pericytes [66]. Overall, although the mechanism by which pericytes control CBF has not been fully elucidated, Hariharan et al. determined a new predictive model for the role of pericyte ion channels and G protein‐coupled receptors (GPCRs) in regulating CBF by combining functional data from pericytes with single‐cell RNA sequencing screening results [78]. Future research can use this tool to explore in depth the molecular mechanisms that control cell physiology of the pericyte, with a view to revealing new mechanisms and targets.

### 4.3 Regulation of Angiogenesis

Studies have shown that in the zebrafish model, pericytes begin to appear 52 h after fertilization (hpf), and during subsequent development, they develop together with the blood vessels of the brain and trunk, indicating that pericytes play an important role in the formation and maturation of blood vessels [79, 80]. In fact, angiogenesis is a process of multicytokine, multicell, and multisignal pathway cascades, which mainly involves the processes of vascular sprouting, vascular stabilization, and vascular maturation. The cascade between pericytes and endothelial cells is considered to be the most important factor in the entire process of angiogenesis. Evidence suggests that a unique pericyte lineage and endothelial cell cascade are generated during angiogenesis to regulate angiogenesis [81]. In addition, during angiogenesis, pericytes secrete VEGF‐A, activate the VEGFR2 pathway on endothelial cells, and promote endothelial cell migration, thereby promoting angiogenesis [82]. Not only that, but the recruitment of pericytes is essential for blood vessel sprouting. At the molecular level, PDGFRβ expressed by pericytes interacts with PDGF‐BB secreted by endothelial cells, causing pericytes to migrate towards endothelial cells [83]. In fact, the link between PDGFRβ and PDGF‐BB also plays an important role during vascular stabilization. Studies have shown that a lack of PDGFR‐β leads to a decrease in pericyte coverage, which in turn leads to vascular instability [84]. Subsequently, during the maturation of blood vessels, multiple signaling pathways are involved, including Ang1/Tie2, TGF‐β/TGF‐βr2, and VEGF and its receptor [85]. Some studies have shown that angiopoietin‐1 is an important factor in the stabilization and maturation of blood vessels. Some studies have shown that under hypoxic conditions, pericytes express angiopoietin‐1, which promotes the stabilization of blood vessels [86]. It is worth noting that pericyte regulates endothelial cell by interacting with the Tie2 receptor after expressing Ang1, while the Tie2 receptor is generally considered to be specifically expressed by endothelial cell [87]. In addition to Ang1/Tie2, Ang2/Tie2 also plays an important role in vascular stabilization and usually antagonizes Ang1/Tie2. In other words, Ang2 mediates pericytes loss and vascular instability [88]. Moreover, the TGF‐β/TGF‐βr2 signaling pathway also plays an important role in the maturation of blood vessels. TGF‐β can mediate the proliferation, differentiation, and survival of pericytes and endothelial cells [89]. Finally, the connection between the endothelial cell and pericytes leads to the deposition of basement membranes and the formation of complete blood vessels to ensure normal blood flow [90].

### 4.4 Regulation of the Immune Response

Pericytes play a significant but often overlooked role in the immune response. It is well known that the transport of leukocytes is coordinated mainly by adhesion molecules and chemokines. However, studies have shown that in an inflammatory environment, pericytes can upregulate the expression of the adhesion molecule ICAM‐1 and release the chemokine MIF to control leukocyte movement [91]. Moreover, LPS treatment of the brain pericyte was found to significantly upregulate the genes CXCL10, CCL2, CXCL8, CCL20, IL‐6, and CXCL1 [92]. These results all indicate that pericytes have the function of controlling the transport of leukocytes. Specifically, mature T cells, as a type of white blood cell, can be regulated by pericytes to enter the bloodstream from the thymus across the endothelium [93]. In addition, previous studies have shown that the migration of neutrophils mainly involves two processes: crossing endothelial cells and crossing pericytes [94]. Proebstl et al. used 3D imaging technology to discover that pericytes are important players in the migration process of neutrophils, and that this process involves the interaction of ICAM‐1 expressed by pericytes with Mac‐1 and LFA‐1 expressed by neutrophils [95]. Besides, in studies using TNF‐α or IL‐1β‐stimulated mouse cremaster muscle, it has been observed that pericytes actively potentiate neutrophil migration. This finding provides evidence that pericytes regulate immune cell migration within neurovascular niches [96]. In addition, pericytes have secretory properties that are crucial for inflammatory immune responses. Pericytes secrete IL‐6 after stimulation with tumor necrosis factor alpha via the IκB‐NFκB and JAK‐STAT3 pathways [97, 98]. Additionally, brain pericytes can express the chemokine CCL11, which suppresses neurogenesis in the aged brain, suggesting a role for brain pericytes in the aging brain [99]. Moreover, after LPS stimulation, brain pericytes secrete interleukin (IL)‐10, IL‐9, IL‐12, granulocyte colony‐stimulating factor, granulocyte‐macrophage colony‐stimulating factor, eosinophil chemotactic factor (CC motif) ligand (CCL)‐3 and CCL‐4 via the MAPK signaling pathway [100]. In addition, pericytes are thought to have certain immunosuppressive functions. Some studies have shown that retinal pericytes can inhibit T cell proliferation and IFN‐γ and TNF‐α secretion and protect ECs from inflammation‐induced apoptosis, suggesting that pericytes have immunosuppressive functions [27]. In addition, human pluripotent stem cell (hPSC)‐derived PCs were found to mediate an increase in the frequency of regulatory T cells (Tregs), which are cells with significant immunosuppressive functions, when co‐cultured with unactivated pericyte blood T cells [101, 102]. This means that under certain circumstances, pericytes can regulate immunosuppression.

**FIGURE 2:**
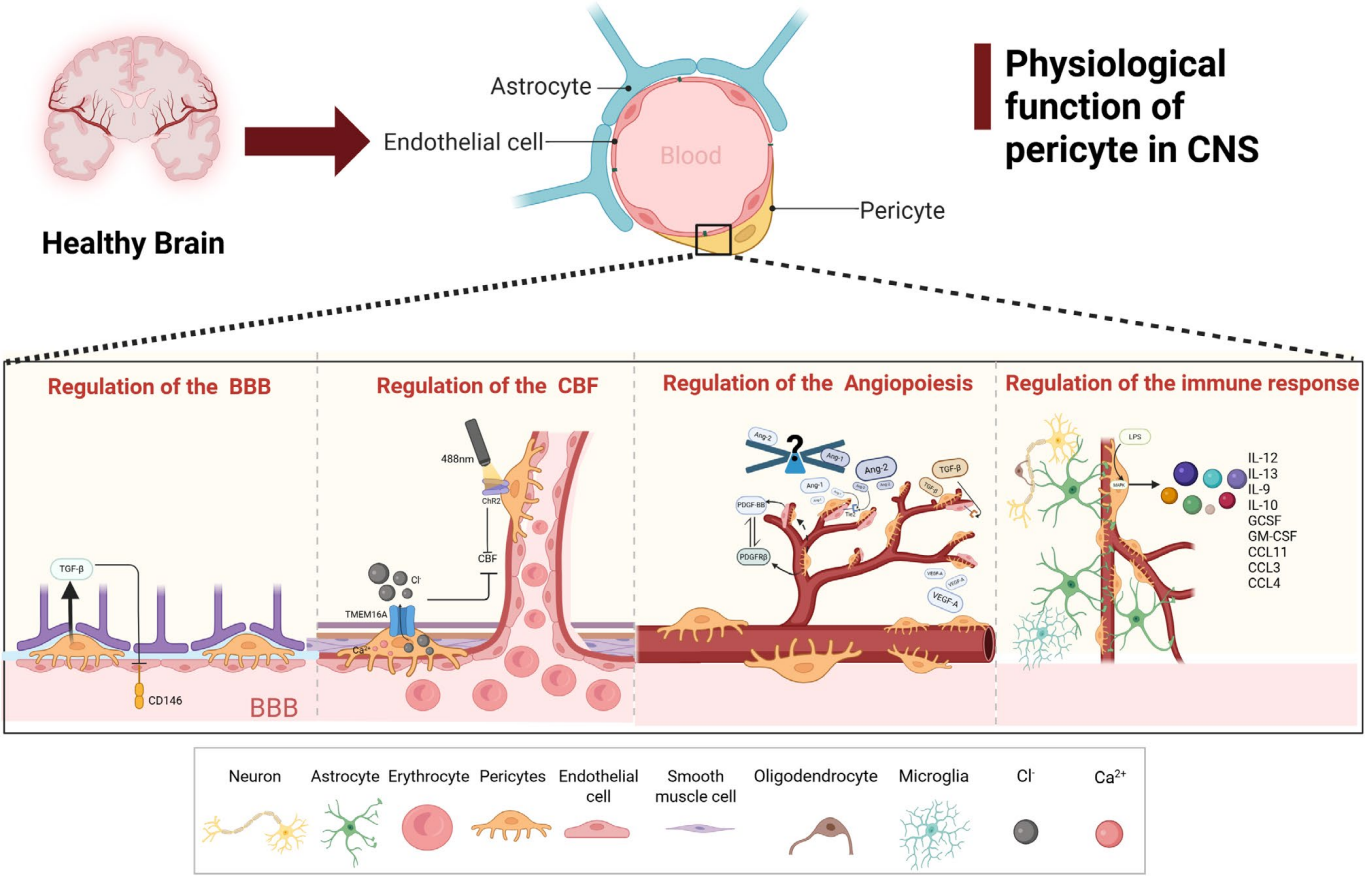
Normal physiological functions of pericytes, such as regulation of the BBB, regulation of cerebral blood flow, regulation of angiogenesis, and regulation of the immune response.

Overall, research on the role of pericyte in the immune response is lacking, but existing studies all indicate that pericytes play an important role in anti‐inflammatory and proinflammatory processes, which means that pericytes may be the key to regulating the inflammatory response (See Figure 2 for details).

## 5 Role of Pericytes in CNS Diseases BBB Dysfunction

Pericytes play a vital role in maintaining CNS homeostasis. We have previously discussed in detail the physiological functions of pericytes. However, in many CNS diseases, dysfunction or loss of pericytes can lead to disruption of the BBB, which in turn can lead to immune cell infiltration, infiltration of inflammatory factors, etc., and thus cause more severe brain damage. For example, in an AD mouse model, pericyte degeneration leads to BBB dysfunction, which allows Aβ accumulation and p‐τ phosphorylation [103]. Moreover, BBB dysfunction associated with pericyte loss has also been observed in AD patients [104]. In addition, a systematic review published in 2022 provides strong evidence for an association between pericyte dysfunction, BBB dysfunction, and AD onset [13]. Moreover, in diseases such as Stroke, AD, and MS, damage to the function of pericytes not only contributes to increased permeability of the BBB but may also aggravate neuroinflammation and oxidative stress. Therefore, in order to gain a deeper understanding of the role of pericytes in CNS diseases, we will discuss in depth below the mechanism of BBB dysfunction in CNS diseases by pericytes, with the aim of providing new ideas for future treatment strategies for CNS diseases that target pericytes (Table 2).

**TABLE 2:** Summary of pericyte and related signaling pathways in CNS diseases.

Diseases	Changes in pericytes	Effects on the body	Key molecules/signaling pathways	Reference
Alzheimer's disease	Pericyte loss, defective PDGFRβ signaling	Inhibited LRP‐1‐mediated Aβ clearance, BBB disruption	ApoE3, LRP1/apoE, CypA‐NF‐κB‐MMP‐9 signaling pathway	[105, 106, 107]
Vascular dementia	Elevated levels of released MMP‐9, PDGFRβ	Tight junction protein degradation, BBB leakage	TGF‐β/Smad2 signaling pathway, TNF‐α	[108, 109, 110, 111, 112]
Multiple sclerosis	P2X7R overexpression, PDGFRβ expression	Immune cell infiltration with increased demyelinating lesions	MHC II	[110, 113, 114]
Ischemic Stroke	Upregulation of PDGFRβ, PDGFβ expression, significant upregulation of NADPH oxidase expression, elevated cytoplasmic Ca2+ concentration, differentiation into fibroblasts, endothelial cells, and microglia	Capillary “no‐reflow phenomenon”, BBB destruction, BBB repair, remodeling, and phagocytosis of necrotic tissue after stroke onset	TMEM16A‐Ca^2+^ channels, ER‐β/miR638/MAPK‐P signaling pathway, NG2+, Tbx18+	[52, 53, 56, 115, 116, 117]
Intracerebral hemorrhage	Decreased expression of platelet‐derived growth factor receptor‐β and NG2, accumulation of high levels of Fe2+ in pericytes, and production of MMP‐9	Exacerbated BBB dysfunction	STAT3, thrombin	[118, 119, 120, 121]

### 5.1 Alzheimer's Disease

Alzheimer's disease (AD) is a common neurodegenerative disease, the main features of which are amyloid beta (Aβ) deposition and the accumulation of intracellular neurofibrillary tangles (NFTS). Previous studies have shown that pericyte loss and vascular platelet‐derived growth factor receptor‐β (PDGFRβ) signaling defects are significant features of BBB dysfunction in AD [35]. An autopsy report showed that the coverage of pericytes in the cerebral cortex and hippocampus of AD patients was reduced by 32% and 33%, respectively, and this phenomenon was associated with increased extravascular immunoglobulin G (IgG) and fibrin deposits. This indicates that the significant loss of pericytes in AD patients is closely related to the severity of BBB degradation [104]. Moreover, ELISA tests have also shown that the pathological characteristics of AD patients are positively correlated with the degree of BBB damage [122]. Interestingly, some studies have shown the opposite: senile (neurofibrillary) plaques are not stained with antialbumin, prealbumin, and fibrinogen antibodies, suggesting that there is no breakdown of the BBB in AD patients [123]. This contradiction may be due to differences in the sample site or the subtype specificity of BBB damage. To solve this problem, future experiments can be conducted on a larger scale to determine the clear mechanism of the pathological process of AD and the dysfunction of the BBB. Aβ protein deposition can lead to neuronal apoptosis, cerebrovascular damage, and activation and proliferation of glial cells. Therefore, this pathological process is considered to play an important role in the pathogenesis of AD [124]. Not only that, but pathological phenomena of Aβ protein deposition and tau protein lesions have also been found in the retinas of AD patients, which is consistent with the changes in the brains of AD patients [125, 126]. The scavenger receptor LDL receptor‐related protein‐1 (LRP‐1) is an endocytic receptor and a member of the low‐density lipoprotein receptor family. It is a key player in regulating the transport of β‐amyloid (Aβ40) across the BBB. However, its specific mechanism in AD has not been fully elucidated, especially the changes in LRP‐1 function at different stages of AD [127]. Studies have shown that downregulation of LRP‐1 and degeneration of pericytes are the main mechanisms mediating BBB dysfunction in patients with AD and animal models of AD [128, 129]. To support this conclusion, an autopsy report observed that in AD patients, the expression of LRP‐1 in retinal blood vessels was reduced, and pericytes had early apoptosis. The early apoptosis of pericytes may have stopped LRP‐1 from clearing Aβ in the AD retina, leading to BBB damage. However, this study did not consider the influence of the genotype of the examinee, especially the human apolipoprotein E (apoE) genotype, and therefore has certain limitations [107]. Previous studies have shown that there are three isoforms of human apolipoprotein E: APOE2, APOE3, and APOE4, and the deletion of APOE4 is the main genetic risk factor for AD [130]. Besides, ApoE3 secreted by astrocytes inhibits the breakdown of the BBB by signaling to pericytes via LRP1 to inhibit the CypA–nuclear factor‐κB–matrix‐metalloproteinase‐9 pathway [105]. Recently, Ma et al. further demonstrated that pericytes clear Aβ deposits through the LRP1/apoE pathway. Notably, silencing apoE3 in mice almost inhibited the clearance of Aβ by pericytes, suggesting that apoE3 mediates the LRP1‐dependent clearance of Cy3‐Aβ42 aggregates rather than apoE4 [106]. This study is the first to propose that LRP‐1 not only acts as a receptor for Aβ clearance, but also participates in the pathological process of AD by regulating the function of pericytes and affecting the homeostasis of the BBB, which is consistent with the results of previous studies. Moreover, these experiments have all demonstrated the importance of APOE genotyping in AD research and treatment. In summary, LRP‐1 expression is downregulated in AD patients, leading to a decrease in Aβ clearance efficiency. Therefore, enhancing the function of LRP‐1 and pericytes is considered a potential strategy for the treatment of AD. Currently, studies have shown that fluvastatin and olive oil extract can increase Aβ clearance by increasing LRP‐1 expression [131, 132]. Although current research on the role of LRP‐1 and pericytes in AD has made some progress, the existing evidence primarily stems from animal studies and has not been proved at the clinical level. Future efforts should focus on designing rigorous human trials to evaluate the therapeutic efficacy of enhancing LRP‐1 expression and modulating pericyte function in AD patients, as well as to determine their potential as clinical treatment targets. It is important to thoroughly assess the specific risks associated with LRP‐1 targeted interventions in APOE4 carriers to refine precision treatment strategies.

### 5.2 Vascular Dementia

VaD is the second most common type of dementia in the world after AD, and CCH is the main pathogenesis of VaD [133]. Long‐term CCH can lead to local ischemia and anoxic microenvironments in the brain, which in turn trigger a series of pathophysiological responses, such as neuroinflammatory responses, BBB dysfunction, and oxidative stress, thereby promoting the occurrence and progression of VaD [134]. An imaging study showed that the degree of BBB breakdown in people with mild cognitive impairment was associated with the levels of PDGFRβ in the CSF. PDGFRβ is a marker of pericyte damage, suggesting that pericytes play an important role in neurodegenerative diseases [135]. Matrix metalloproteinase (MMP) is a zinc‐dependent protein hydrolase that can damage the BBB by degrading TJPs [136, 137]. Recent studies have shown that pericytes, as an important component of the BBB, are the main source of MMP‐9, and that TNF‐α is key to inducing the release of MMP‐9 from pericytes [108, 109]. A significant increase in MMP‐9 levels in the serum of VaD patients was observed in a clinical trial [138]. Therefore, we assume that BBB dysfunction in VaD is mainly mediated by pericytes. However, the mechanism by which pericytes release MMP‐9 to mediate BBB damage is not yet clear. The current views mainly focus on the degradation of BMEC extracellular matrix and tight junction proteins or the loss of pericytes. In addition, in experimental CCH mice, the coverage of pericytes in the corpus callosum is reduced, and BBB permeability is increased [139]. Moreover, experiments have further shown that BBB dysfunction caused by reduced pericyte coverage mainly leads to brain damage by regulating the TGF‐β/Smad2 signaling pathway [140]. In addition, CCH also promotes the production of reactive oxygen species (ROS), which can cause serious damage to the BBB by activating MMPs and oxidizing cell molecules. However, it is still debatable whether this process involves pericyte contraction [141]. Hirunpattarasilp et al. found that increased ROS levels in the cytoplasm and mitochondria are not involved in pericyte‐mediated capillary contraction [112]. However, Liu et al. found that the loss of contractile ability of pericytes in diabetic patients is associated with enhanced ROS and reduced ATP production, a process that mediates the breakdown of the BBB [111].

Therefore, these pathological processes do not develop independently of each other during the pathogenesis of VaD, but rather mutually promote each other. However, the role of pericytes in these pathological processes (especially BBB dysfunction) cannot be generalized and still needs to be further explored.

### 5.3 Multiple Sclerosis

MS is an autoimmune disease characterized by demyelination, dysfunction of pericytes, dysfunction of the BBB, death of oligodendrocytes, and damage to axons. However, the pathogenesis of MS is still unclear [142, 143]. Chondroitin sulfate proteoglycan (CSPG), a marker of pericytes, has been shown to significantly inhibit OPC development and thus myelin regeneration during the pathogenesis of MS [144]. In addition, studies have further shown that pericytes mediate macrophage infiltration after CSPG stimulation, but pericytes were not detected in the brain parenchyma of MS [145]. This is contrary to the migratory properties of pericytes in CNS diseases, which suggests a different role for pericytes in different CNS diseases. Moreover, studies have shown that pericytes can present antigens via MHC II molecules, promote T cell proliferation and activation, and in the EAE mouse model (a model of MS with the characteristics of the pathogenesis of human MS), it was observed that after pericyte MHC II depletion, CD4 + T cell infiltration in the CNS was reduced [146]. It can therefore be speculated that pericytes play an important role in the neuroinflammation of MS, mainly assisting the recruitment of monocytes, T cells, eosinophils, and neutrophils, which leads to more severe demyelination and blood–brain barrier dysfunction.

Moreover, a large number of studies have proven that BBB dysfunction is a prominent feature of MS. BBB dysfunction was found in a mouse model of MS and is the earliest pathological change to occur in the development of MS [147]. In addition, evidence shows that the loss of endothelial tight junction proteins is found in brain specimens obtained from MS patients, which indicates the occurrence of BBB dysfunction in the progression of MS [148]. It is noteworthy that BBB dysfunction can lead to the entry of activated immune cells into the CNS, which is consistent with the pathological events of immune cell infiltration in MS. In the experimental autoimmune encephalomyelitis (EAE) mouse model, overexpression of P2X7R, a receptor that mediates the activation of inflammatory bodies and the release of interleukin‐1β (IL‐1β), was observed in pericytes. The expression of PDGFRβ and claudin‐5 was increased after the use of P2X7R antagonists, further illustrating the secretory properties of pericytes in MS [110, 149]. An autopsy report on MS patients showed that with the progression of the MS disease process, the expression of PDGFRβ, a marker of pericytes, was upregulated in areas with brain lesions, suggesting a link between pericytes and the progression of MS [113]. In addition, as the MS progresses, demyelination also worsens, and this process is related to pericyte and OPC. Studies have shown that the development of myelin sheaths mainly depends on the interaction between pericytes and oligodendrocytes, and the absence of NG2 in OPC and pericyte can further lead to remyelination and restore BBB function [114, 150]. Not only that, but the demyelination process in MS produces a large number of myelin fragments, which in turn further inhibit remyelination. Whether pericytes, a type of cell with phagocytic properties, are the key to recovery from MS remains to be discussed [151].

In summary, MS is an autoimmune disease in which pericytes may be an important mediator of the neuroinflammatory response in MS, leading to more severe demyelination and BBB dysfunction. Therefore, limiting the passage of inflammatory immune cells and inhibiting the secretion of inflammatory factors may be one of the future strategies for the treatment of MS.

### 5.4 Ischemic Stroke

**FIGURE 3:**
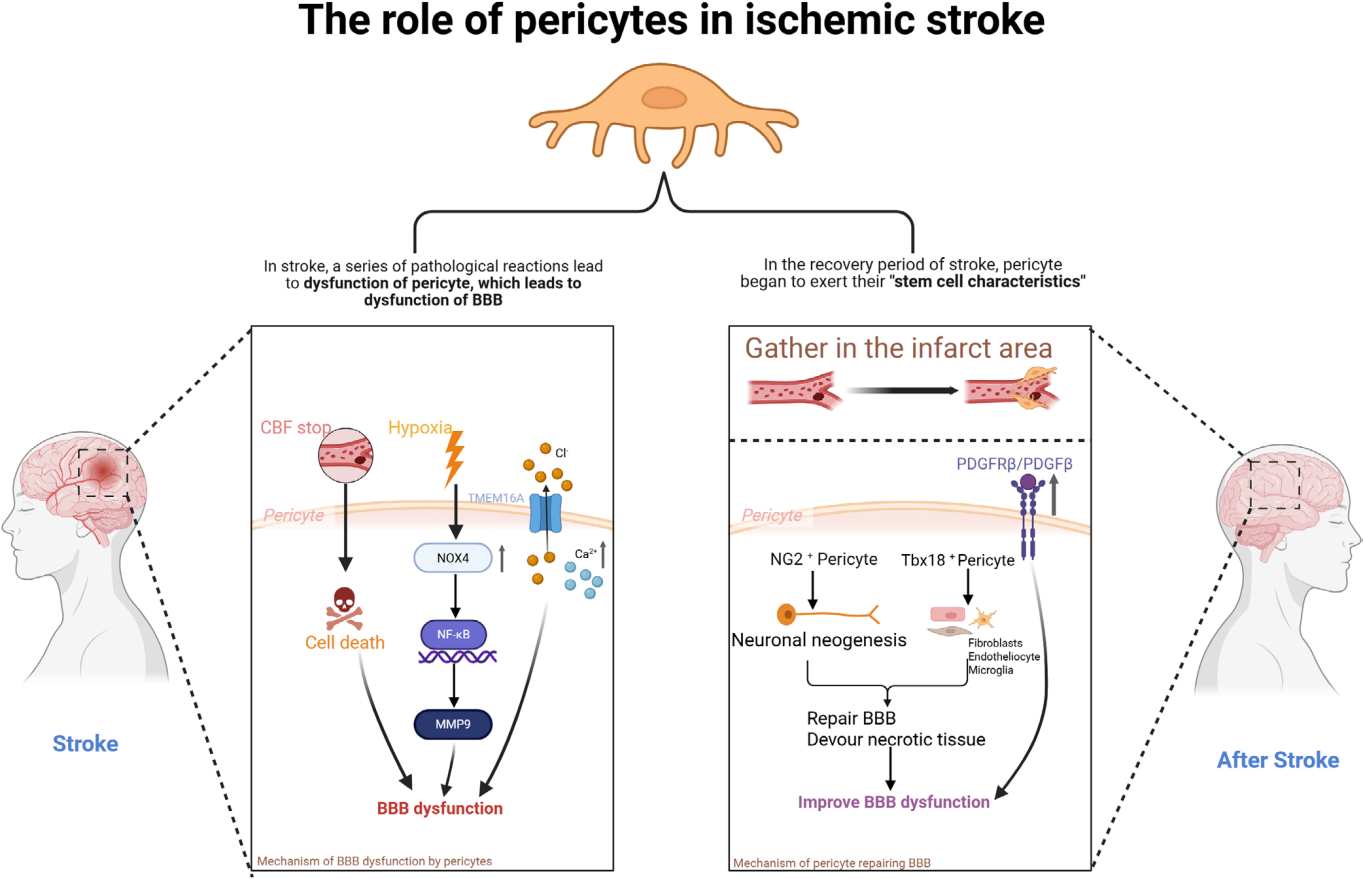
The role of pericytes in ischemic stroke.

IS is a disease caused by interruption or reduction of blood supply to the brain, leading to hypoxia and necrosis of brain tissue [152]. However, as a key component of the BBB, pericytes seem to play a double‐edged role in the process of IS. In the early stages of blood supply stop, pericytes die earlier than other cells in the NVU, and this death can lead to the loss of TJs, which can lead to the breakdown of the BBB [68]. Moreover, experiments have confirmed that the loss of function of hypoxia‐inducible factor‐1 (HIF‐1) in pericytes can reduce ischemic brain injury and BBB dysfunction [153]. Furthermore, the activation of matrix metalloproteinases (MMPs) within the cytoplasmic compartment of pericytes accelerates the proteolytic degradation of BBB constituents, leading to capillary structural compromise and exacerbation of BBB dysfunction [117]. Moreover, in MCAO (middle cerebral artery occlusion, a model that can simulate human cerebral ischemia) mouse, NADPH oxidase 4 (NOX4) expression was significantly upregulated in the pericytes surrounding the infarct tissue, and further destroyed the BBB by enhancing MMP‐9 activity [154]. In addition, we have previously described that TNF‐α can induce the release of MMP‐9 from pericyte and can induce the migration of pericyte [109]. Recently, studies have shown that estrogen can improve BBB damage after ischemic stroke by inhibiting TNF‐α‐induced pericyte migration through the ER‐β/miR638/MAPK‐P signaling pathway [115]. Furthermore, a transcriptomic analysis focusing on estradiol‐regulated genes in pericytes has further corroborated this concept, providing robust molecular evidence for the proposed mechanism [116]. Not only that, after stroke, the elevated cytoplasmic Ca2+ concentration in pericytes activates the Ca2+ gated anion channel TMEM16A, causing chloride ions to flow out, the cell membrane to depolarize, and voltage‐gated calcium channels to open, resulting in strong contraction of the pericyte and aggravating the BBB dysfunction, leading to the “no‐reflux phenomenon” after stroke. These studies have shown that after stroke, pericytes damage the CNS [73]. Interestingly, after stroke, pericytes begin to exert their “stem cell‐like” properties to repair the BBB, remodel blood vessels, and phagocytize necrotic tissue. The accumulation of pericytes and upregulation of PDGFRβ and PDGFβ expression in the infarct area after stroke can be observed, suggesting that these cells promote angiogenesis and BBB repair after stroke. These cells have been identified as cells derived from bone marrow (BMDC) [155, 156]. Not only that, after stroke, pericytes will leave the blood vessel wall and proliferate and differentiate into microglia, which play a beneficial role in phagocytizing necrotic tissue and cells [157, 158]. In addition, single‐cell analysis of pericyte after stroke revealed that NG2^+^ pericyte express a dominant neural reprogramming potential to generate new neurons, while Tbx18^+^ pericyte exhibit strong repair capabilities by generating fibroblasts, endothelial cells, and microglia after IS [159]. These results all suggest the stem cell properties of pericyte in IS. In the future, the specific stem cell properties of pericytes after IS can be further explored, and research on how to regulate the proliferation and differentiation of pericytes after stroke may provide a new direction for clinical treatment (See Figure 3 for details).

### 5.5 Intracerebral Hemorrhage

ICH, a type of stroke, is the second most common subtype of stroke and is characterized by a high morbidity and mortality rate. The main pathological change in secondary brain injury after ICH is BBB dysfunction, which can lead to severe perihematomal edema and immune response [160]. In ICH, the expression of platelet‐derived growth factor receptor‐β and NG2 derived from pericytes is reduced, which indicates the loss of pericytes. However, Leptin improves this phenomenon and promotes angiogenesis after ICH, and this process is mainly involved in the signal pathway of transcription activator 3 (STAT3) [118]. Notably, in a collagenase‐induced ICH mouse model, the administration of cilostazol improved pericyte loss and thus BBB dysfunction, providing strong evidence that pericytes are key cells in BBB dysfunction in ICH [119]. Moreover, oxidative stress after ICH also worsens BBB dysfunction. Some studies have shown that after ICH, pericyte cells accumulate a large amount of Fe^2+^ and die, which leads to BBB damage [161]. However, the relationship between thrombin and pericytes is not yet clear. Some studies have shown that thrombin increases the phosphorylation of Tie2 and Akt, the expression of PI3K and the coverage of pericytes, i.e., pericytes can survive in thrombin and maintain the BBB [120]. However, some studies have shown that thrombin induces perivascular cells to produce MMP‐9, which causes BBB dysfunction and aggravates brain injury [121].

In general, the changes in the pericyte as a potential target for the treatment of ICH may involve multiple mechanisms and still need to be further explored.

## 6 Treatment Based on Pericytes

### 6.1 Regenerative Medicine That Induces Differentiation of Pericytes

Pericyte is a cell that has the characteristics of a stem cell and can differentiate into different types of cells in vitro and in vivo, such as macrophages, vascular smooth muscle cells, osteoblasts, chondrocytes, cardiomyocytes, astrocytes, neurons, etc. [158, 162, 163, 164, 165]. Studies in the CNS have demonstrated that brain pericytes can serve as neuronal progenitors, and their reprogramming into neuronal cells primarily requires retrovirus‐mediated co‐expression of transcription factors Sox2 and Mash1 [166]. However, we should also be aware of the limitations of existing research. Studies based on the stem cell properties of pericyte cells are all in vitro studies. An in vivo study showed that T‐Box transcription factor + (Tbx18+) pericyte and vascular smooth muscle cells do not exhibit differentiation potential in the aging and pathological environment [167]. Interestingly, under ischemic/hypoxic conditions, pericyte can acquire multipotent stem cell activity and can differentiate into the main components of BBB [168]. Contrary to prior findings, this suggests that the stem cell properties of pericytes in the CNS may be regulated by diverse mechanisms. However, the stem cell characteristics of pericytes do not always confer benefits. After renal ischemia–reperfusion, inflammatory cytokines and chemokines are released into the renal interstitium, stimulating the proliferation of mesangial cells and the production of extracellular matrix (ECM), leading to renal fibrosis [169]. Notably, during renal fibrosis, pericytes differentiate into myofibroblasts, thereby exacerbating the process. But this process can be inhibited by paracrine signaling from Exogenous bone marrow derived‐putative endothelial progenitor cells (b‐pEPCs), although the underlying mechanisms remain unclear [170, 171].

Additionally, given the potential of mesenchymal stem cells (MSCs) to replace or repair pericytes, studies have shown that MSCs, after injection into the carotid artery, can migrate to brain injury sites, potentially replacing damaged pericytes. However, during this process, MSCs may be limited in their effectiveness in nerve injury repair due to low survival rates and rejection by damaged brain tissue [172]. Nevertheless, PDGFR‐β + MSCs and differentiated mesenchymal cells (DMCs) exhibit significant proangiogenic properties, suggesting the strong potential of MSCs in replacing or repairing pericytes [173, 174]. In conclusion, these studies provide some interesting directions for researchers: further exploration is needed on how to use MSCs to replace or repair damaged pericytes, or how to deeply research the stem cell characteristics of pericytes themselves. Nevertheless, it is evident that the stem and pericyte's stem characteristics provide novel perspectives for neurorepair and cerebrovascular pathologies. Future studies should explore the stem function of pericytes and elucidate the relationship between pericytes and the stem cells.

### 6.2 Pericyte‐Extracellular Vesicles (PC‐EVs)

There are still many difficulties in the treatment of CNS diseases, which are mainly reflected in the inability of drugs to cross the BBB to reach the CNS. However, PC‐EVs offer hope for a solution to this problem. PC‐EVs are EVs with a diameter of 30 to 350 nm, a bilayer structure, and a spherical or cup‐shaped morphology. They express markers such as CD9 and CD81 [4]. Not only that, PC‐EVs have powerful secretory functions and mainly rely on EVs to release cytokines. For example, a proteomics analysis of PC‐EVs found that Ang‐1 is present in the vesicles, indicating that PC‐EVs have a certain protective effect on the BBB [175]. In addition, PC‐EVs carry a variety of cytokines. Research indicates that brain PC‐EVs carry growth factors such as vascular endothelial growth factor (VEGF), fibroblast growth factor (FGF), heparin‐binding EGF‐like growth factor (HB‐EGF), brain‐derived neurotrophic factor (BDNF), neurotrophin 3 (NT3), nerve growth factor (NGF), vascular endothelial growth factor (VEGF), and insulin‐like growth factor‐binding protein (IGFBP) [176]. In pathological conditions, the cytokines secreted by pericyte are mainly dependent on environmental conditions. Some studies have shown that under the stimulation of LPS, PC‐EVs in the brain mainly carry inflammatory cytokines, such as IL‐8, IL‐10, IL‐6, and MCP‐1 [177]. Moreover, PC‐EVs reduced the expression of HIF‐1α, Bax, aquaporin‐4, and MMP2, increased the expression of Claudin‐5 and Bcl‐2, and inhibited apoptosis in a spinal cord injury (SCI) model mouse [178]. PC‐EVs also carry microRNAs (miRNAs). The types of miRNAs currently identified are miR‐143, miR26a, miR122‐5p, miR21, miR181a, etc. These miRNAs have been shown to play a role in diseases such as cerebrovascular disease, atherosclerosis, and neurodegenerative diseases [179, 180, 181, 182]. The mechanism of action of PC‐EVs, a new treatment, has not been elucidated, and EVs do not always show benefits. In neurodegenerative diseases, EVs may carry misfolded and mutant proteins, which may aggravate the progression of the disease [183]. However, we firmly believe that in‐depth research on the PC ‐ EVs will enable researchers to better understand the mechanism of the pericyte and develop new clinical applications.

## 7 Progress in Clinical Trial

In the current field of clinical trials, researchers often associate pericytes with tumors, mainly because of the strong link between pericytes and angiogenesis [156]. Bevacizumab has been shown to result in a significant increase in pericyte coverage in tumor biopsies from patients with locally advanced nasopharyngeal carcinoma [184]. In addition, studies have shown that imatinib, as a PDGFRβ inhibitor, can inhibit the differentiation of pericytes into fibroblasts by blocking the PDGF‐BB/PDGFRβ signaling pathway, thus improving pulmonary fibrosis [185]. In the CNS, imatinib can also promote axonal regeneration and reduce fibrotic scar formation after SCI by blocking the PDGF‐BB/PDGFRβ signaling pathway [186]. Therefore, it is also an interesting idea to prevent pericytes from differentiating into fibroblasts to improve fibrosis, but this view has not been confirmed in clinical trials. In a recent phase 2 study of thalidomide in the treatment of radiation‐induced blood–brain barrier damage, thalidomide was found to restore BBB function as well as cerebral perfusion. This positive effect can be attributed to the upregulation of platelet‐derived growth factor receptor‐β (PDGFRβ) expression by thalidomide, implying the rescue of pericyte function [187]. This promising result indicates the importance of pericytes in the treatment of BBB. In addition, with the emergence of stem cell therapy, using stem cells to replace the pericytes to improve BBB dysfunction is also a valuable research approach. Specifically, thanks to the discovery of pericyte‐like cells derived from cranial nerve crest cells (hPSC‐CNPC PCs), studies have shown that intravenous injection of hPSC‐CNPC PCs into MCAO mice can improve BBB dysfunction and prevent neuronal apoptosis [188]. Therefore, hPSC‐CNPC PCs may be an ideal cell source for cell therapy in CNS diseases associated with BBB dysfunction. However, the optimal administration method for this therapy, as well as its safety and efficacy, still needs further clarification.

However, there are currently few studies focusing on pericytes, and in addition, a search of ClinicalTrials.gov for the keyword pericyte (accessed March 12, 2025) identified 33 ongoing studies; however, almost none of these focused on CNS diseases, a situation that suggests a large unexplored gap in the field of pericytes in the treatment of CNS diseases. Therefore, there is a need to focus future research on pericytes to delve deeper into their potential value in the treatment of CNS diseases.

## 8 Conclusions

As key components of the BBB and NVU, pericytes play an important role in maintaining the stability of the CNS by regulating physiological functions such as BBB function, CBF, angiogenesis, and immune responses. This review summarizes the biological characteristics of pericytes and their pathological mechanisms in AD, VaD, MS, IS, ICH, and other CNS diseases. Studies have shown that pericyte dysfunction or loss is a core factor in BBB damage, leading to pathological reactions such as neuroinflammation, immune cell infiltration, and oxidative stress, which in turn exacerbate brain damage. For example, in AD, the apoptosis of pericytes and the downregulation of LRP‐1 signaling together reduce the clearance of Aβ, while abnormal contraction or death of pericytes after IS aggravates the “no‐reflow phenomenon”. It is worth noting that pericytes exhibit functional heterogeneity in different diseases, and their role may be dynamically regulated by developmental origin, anatomical location, and microenvironmental signals. However, the regulatory mechanism is still controversial. For example, whether pericytes regulate CBF independently of smooth muscle cells still requires further research. Future studies should focus on clarifying the molecular characteristics of different pericyte subtypes and their role in BBB regulation and immunomodulation, revealing the relationship between pericytes and endothelial cells as well as astrocytes. Additionally, research should explore the clinical application potential of pericytes in regenerative medicine, such as replacing diseased pericytes through stem cell therapy, or inhibiting the differentiation of pericytes into fibroblasts to improve fibrosis. Another direction could involve inducing pericytes to differentiate into other cell types to repair damaged tissues, or regulating the delivery of neurotrophic factors through extracellular vesicles. The safety and efficacy of targeting TMEM16A ion channels or LRP‐1 signaling should also be evaluated. In summary, pericytes offer a new perspective for understanding the mechanisms of CNS diseases and open up new avenues for developing neuroprotective therapies that overcome the BBB. In the future, interdisciplinary collaboration should be strengthened to promote the translation of basic discoveries into clinical practice and achieve precision therapies targeting pericytes.

## Author Contributions

Conception and design: Zhiying Chen, Tianrui Yu. Administrative support: Zhiying Chen, Zixuan Wang, and Yuanyuan Xiang. Provision of study materials: Moxin Wu, Xiaoping Yin. Collection and assembly of data: Moxin Wu, Xiaoping Yin. Data analysis and interpretation: Zhiying Chen, Zixuan Wang. Manuscript writing: All authors. Final approval of manuscript: All authors. The final version has been revised by: Zhiying Chen.

## Ethics Statement

The authors have nothing to report.

## Consent

The authors have nothing to report.

## Conflicts of Interest

The authors declare no conflicts of interest.

## Data Availability

The datasets used and/or analyzed during the current study are available from the corresponding author on reasonable request.
